# Differences in occupational values, communication types, job satisfaction, and organisational commitment among clinical nurses across generations

**DOI:** 10.3389/fpsyg.2023.1174197

**Published:** 2023-07-14

**Authors:** Seul A. Lee, Jungmin Lee

**Affiliations:** ^1^Department of Nursing Education, Hallym University, Chuncheon-si, Gangwon-do, Republic of Korea; ^2^School of Nursing, Hallym University, Chuncheon-si, Gangwon-do, Republic of Korea

**Keywords:** age distribution, communication, nurses, job satisfaction, organizations

## Abstract

**Introduction:**

This study examined occupational values, communication types, job satisfaction, and organisational commitment among clinical nurses across generations.

**Methods:**

Participants were 159 clinical nurses. Data were collected from 1 to 15 August, 2022, using a self-reported questionnaire.

**Results:**

Statistically significant differences were observed across generations in terms of job satisfaction (*F* = 3.492, *p* = 0.03) and organisational commitment (*F* = 4.371, *p* = 0.01). However, there were no differences in occupational values (*F* = 0.765, *p* = 0.47) or communication types (*F* = 1.744, *p* = 0.18) among clinical nurses across generations. There was a moderate to strong positive correlation between job satisfaction and organisational commitment across all generations, and a moderate positive correlation between job satisfaction and occupational values among Generations Y and Z.

**Discussion:**

To improve the quality of nursing care, various intervention programs based on the generational gap among clinical nurses need to be developed, to reduce conflicts, and ultimately establishing the necessary systems through mutual understanding of education.

## 1. Introduction

Generational problems are increasing in South Korea as the country undergoes rapid societal change (Jeon, 2018). The social and cultural backgrounds of Generations X, Y, and Z vary, as do their values, attitudes, growth, and societal problems. Conflicts may arise between younger generations, who refuse to conform to strict and vertical organisational cultures, even when offered high compensation or social recognition, and older generations, who prioritise compensation and social recognition over leisure (Goh et al., 2021). Differences in occupational values and communication types may fuel these conflicts, which may destroy or disrupt organisational culture, consequently affecting occupational performance and satisfaction, and increasing turnover rates.

Hospitals have diverse occupations and strata. Staff members often experience conflict when they actively interact and work with one another within the unique and complex organisational culture and structure of hospitals (Kwon et al., 2019). One study reported that Korean nurses have trouble forming relationships with co-workers and adapting to the hospital environment. Nurses face many challenges as they work across multiple departments, handle several patients or their guardians, and experience emotional labour in an authoritative work culture, which may be perceived as unfair by younger generations (Chae and Kim, 2022).

Generational problems within an organisation are affected by a multitude of factors, including the organisation’s situation and structure, hierarchical relationships, age, and work experience, which may not be immediately apparent. The ages of nurses range between 20 and 50 years, and nurses from different generations have diverse nursing practices and attitudes (Choi and Kim, 2014). Studies have reported generational differences in nursing practices, work attitudes, and values among nurses (Choi and Kim, 2014) as well as differences in communication types, which can lead to a lack of communication at work (Jeon and Lee, 2017). Younger generations have lower work satisfaction and organisational commitment levels and higher turnover intentions than older generations, indicating the importance of examining generational differences in values in an organisation and the need to change workplace management practices that were geared toward older generations (Oh, 2019).

Occupational values affect not only attitudes and decision-making at work but also work and life satisfaction (Seo et al., 2003; Mastracci et al., 2011). Differences in occupational values can entail differences in attitudes, decision-making, interests, and desires related to work, thus contributing to generational conflicts within an organisation.

Studies have identified communication skills as an important resource for reducing nurses’ emotional labour. While effective communication increases nursing performance and reduces conflict in a team, 56.4% of nurses reported communication as their biggest challenge, indicating the need to improve communication skills among nurses (Seo et al., 2003; Im et al., 2012). Additionally, differences in communication types attributed to age diversity in the workplace can lead to communication challenges, thereby negatively affecting nursing performance (Chae and Kim, 2022).

Job satisfaction among nurses positively affects their performance and leads to patient satisfaction, thereby improving the performance of the hospital as a whole (Kim and Kang, 2016). Job satisfaction increases motivation to work, promotes self-growth, and encourages voluntary participation and creativity to reach organisational goals (Park and Kim, 2010), thereby maximising organisational efficiency. Therefore, from a hospital administrations’ perspective, it is important to effectively manage the nursing workforce by hiring excellent nurses and retaining experienced and competent nurses (Cheong and Yun, 2013).

Organisational commitment refers to one’s willingness to work for an organisation for a sustained period based on the individual’s positive views about its’ goals and values; this is an important metric for organisational efficacy and productivity (Mowday et al., 1979). Organisational commitment motivates nurses to work harder, reduces turnover rates, and is closely associated with their work attitudes. These factors improve nursing performance and quality of nursing care received by patients (Lee and Jung, 2019). Organisational commitment is meaningful to both the organisation and its members. Members receive extrinsic rewards and psychological satisfaction through organisational commitment, and the organisation benefits from committed members who are less likely to be absent from work or move to another organisation and are likely to perform well (Mowday et al., 1979).

Few studies have examined generational differences in occupational values, communication types, job satisfaction, or organisational commitment among healthcare workers. Therefore, generational differences in values, attitudes, and behaviours must be investigated. The results of this study may be used as basic data to explore methods for reducing generational conflict and improving organisational efficacy by helping nurses across generations come together to effectively manage the medical workforce.

## 2. Materials and methods

This descriptive survey study aimed to examine the relationships between occupational values, communication types, job satisfaction, and organisational commitment across generations to develop methods for managing the nursing workforce, reducing turnover rates, and improving the quality of nursing care.

### 2.1. Subjects

The study participants were 160 clinical nurses working at H University Hospital, Seoul, which has 641 wards. Nurses from different generations who provided direct care to patients and consented to participate in this study after being informed of its purpose were recruited using quota sampling. On a questionnaire, they were asked to mark the generation to which they belonged to Generation X (1964–1978), Generation Y (1979–1992), or Generation Z (1993–1999).

The sample size was calculated using G-Power 3.1.9.4. It was determined to be 143, given an effect size of 0.25, significance level (α) of 0.05, and power (1-β) of 0.90. Considering dropout and incomplete response rates of 10%, 160 nurses were recruited. One questionnaire with incomplete responses was excluded, and the remaining 159 were used in this study.

### 2.2. Research tools

#### 2.2.1. General characteristics

The questionnaire used in this study contained 18 questions related to general characteristics, including four on sociodemographic characteristics, six on work life, and eight on generational characteristics.

#### 2.2.2. Occupational values

A version of the Maryland Work Value Inventory developed by Mietus (1977), translated by Ahn and Lee (1998), and revised by Han (2014) was used to investigate occupational values. The tool contains eight questions: four on extrinsic values (compensation, reputation or status, financial stability, and work environment) and four on intrinsic values (skills development, personal satisfaction, altruism toward society, and achievement and personal interest). Each question was rated on a five-point Likert scale, with one point given for “Not true at all” and five points for “Very true”. The tool had a Cronbach’s α of 0.81 in a study by Han (2014) and 0.72 in this study.

#### 2.2.3. Communication types

A version of the Rhetorical Sensitivity Scale developed by Hart et al. (1980) and adopted by Lee (2007) and Park and Lee (2013) was used to investigate generational differences in communication types. Questions were rated on a five-point Likert scale, with one point given for “Not true at all” and five points for “Very true”. In previous studies, the tool had a Cronbach’s α of 0.75, and 0.71 in this study.

#### 2.2.4. Job satisfaction

The tool used by Lee et al. (2018) was used to measure job satisfaction with permission from the authors. The tool contains 33 questions across six domains: organisational acknowledgement and professional achievement (nine questions), personal professional growth (six questions), respectful interpersonal relationships (eight questions), completing duty as a nurse (four questions), applying professional skills (three questions), and job security and sense of worth from work (three questions). Each question is rated on a five-point Likert scale, with five points given for “Very true”, four for “True”, three for “Neutral”, two for “Not true”, and one for “Not true at all”. The total score ranged from 33 to 165 points. Higher scores indicate higher job satisfaction. The tool had a Cronbach’s α of 0.95 at the time of its development by Lee et al. (2018) and 0.92 in the current study.

A version of the tool developed by Chang and Chang (2009) to measure organisational commitment among nurses from university hospitals in Taiwan, translated by Choi and Lee (2012), was used in this study. The tool contains 12 questions across the following domains: value (four questions), effort (four questions), and continuance commitment (four questions). Each question was rated from one to five, and the total score ranged from 8 to 40 points. Higher scores indicate higher organisational commitment. The tool had a Cronbach’s α of 0.88 in a study by Choi and Lee (2012) and 0.87 in the current study.

### 2.3. Data collection

Questionnaires were collected from clinical nurses who worked at H University Hospital, Seoul, from August 1, 2022 to August 15, 2022 following the approval of the Institutional Review Board of H University (HIRB-2022-048). The nurses were provided with an information sheet that included information about the purpose of the study, confidentiality, freedom to withdraw from participation, and the principal investigator. The questionnaires were self-reported and sealed in an envelope along with a signed consent form. The researchers visited each department to retrieve the questionnaires.

### 2.4. Data analysis

Data were analysed using SPSS Windows version 21.0.

### 2.5. Ethical considerations

Before completing the questionnaire, respondents were informed about the study’s purpose and process, and that all collected data would be used for academic purposes only. The respondents were included only if they had been provided with an information sheet about the purpose of the study and the content of the questionnaire, and if they consented to participate. They were informed about the benefits of study participation, its voluntary nature, and that there would be no consequences if they opted out. They were informed that all personal information would be used for identification purposes and anonymised during the analysis. For data protection purposes, personally identifiable information was not included in the questionnaire.

## 3. Results

### 3.1. General characteristics

A total of 159 participants (42 nurses from Generation X, 63 from Generation Y, and 54 from Generation Z) were included in the study. The general characteristics of the nurses are presented in Table 1.

**Table 1:** General characteristics of participants (*N* = 159).

Characteristics	Categories	X generation (*n* = 42)	Y generation (*n* = 63)	Z generation (*n* = 54)	*t* or *F*	*p*
		*N* (%)	*N* (%)	*N* (%)		
Age	M ± SD (min-max)	47.43 ± 2.7 (44–57)	35.57 ± 3.8 (30–43)	25.65 ± 1.7 (23–29)		
Marital status	Married	38 (90.5)	38 (60.3)	2 (3.7)	72.28	<0.001
	Unmarried	4 (9.5)	25 (39.7)	52 (96.3)		
Sex	Male	–	3 (4.8)	9 (16.7)	5.56	0.005
	Female	42 (100)	60 (95.2)	45 (83.3)		
Monthly income	Under 250	1 (2.4)	–	2 (3.7)	119.80	<0.001
	250–300	–	2 (3.1)	11 (20.4)		
	300–350	–	19 (30.2)	40 (74.1)		
	Above 350	41 (97.6)	42 (66.7)	1 (1.8)		
Year of issuance of the license	M ± SD (min-max)	1997.24 ± 3.2 (1986–2002)	2009.05 ± 4.1 (2001–2019)	2019.04 ± 3.9 (2001–2022)		
Working department	Medicine	6 (14.3)	16 (25.4)	14 (25.9)	0.374	0.690
	Surgery	6 (14.3)	6 (9.5)	9 (16.7)		
	Outpatient	16 (38.1)	7 (11.1)	6 (11.1)		
	OR	4 (9.5)	15 (23.8)	10 (18.5)		
	ER	4 (9.5)	7 (11.1)	12 (22.2)		
	ICU	3 (7.1)	10 (15.9)	3 (5.6)		
	Others	3 (7.1)	2 (3.2)	–		
Work type	3rd shift	10 (23.8)	52 (82.5)	51 (94.4)	41.69	<0.001
	2nd shift	9 (21.4)	1 (1.6)	1 (1.9)		
	Full-time	23 (54.8)	10 (15.9)	2 (3.7)		
Position	Nurse	16 (38.1)	63 (100)	54 (100)	85.57	<0.001
	CN	25 (59.5)				
	HN	1 (2.4)				
Work–life priorities	High salary	10 (23.8)	32 (50.8)	26 (48.1)	2.65	0.074
	Relationship	18 (42.9)	12 (19.0)	17 (31.5)		
	Holiday	4 (9.5)	4 (6.3)	7 (13.0)		
	Performance	7 (16.7)	5 (7.9)	3 (5.6)		
	Regular working hours	3 (7.1)	10 (15.9)	1 (1.9)		
Total clinical experience (month)	M ± SD (min-max)	287.55 ± 36.3 220–400	154.11 ± 48.8 (36–254)	28.63 ± 18.1 (0–5)		
Current department experience (month)	M ± SD (min-max)	98.98 ± 101.2 (1–301)	84.24 ± 67.7 (3–254)	26.04 ± 17.7 (2–64)		
Felt a generation gap	Not at all	–	2 (3.2)	4 (7.4)	25.68	<0.001
	Not really	3 (7.1)	15 (23.8)	25 (46.3)		
	Sometimes	20 (47.6)	38 (60.3)	24 (44.4)		
	All ways	19 (45.3)	8 (12.7)	1 (1.9)		
Conflict over generation gap	Not at all	1 (2.4)	7 (11.1)	2 (3.7)	15.37	<0.001
	Not really	14 (33.3)	11 (17.5)	13 (24.1)		
	Sometimes	21 (50.0)	25 (29.7)	9 (16.7)		
	Always	3 (7.1)	3 (4.8)	1 (1.9)		
Types of generation gap conflict	Way of thinking	15 (35.7)	19 (28.6)	5 (9.3)	3.80	0.025
	–Work–life balance	–	3 (28.6)	1 (1.9)		
	Conversation topic	–	1 (1.6)	–		
	Conversational style	6 (14.3)	4 (6.3)	1 (1.9)		
	Way of working	–	1 (1.6)	2 (3.7)		
	Work environment	3 (7.1)	1 (1.6)	1 (1.9)		
Negative impact of the generation gap	Yes	13 (31.0)	25 (39.7)	10 (18.5)	11.52	<0.001
	No	26 (61.9)	21 (33.3)	16 (29.6)		
If yes, the reason	Decline in motivation	–	7 (11.1)	3 (5.6)	2.94	0.056
	Turnover intention	–	3 (4.8)	1 (1.9)		
	Lack of communication	9 (21.4)	7 (11.1)	4 (7.4)		
	Reduced concentration	3 (7.1)	4 (6.3)	1 (1.9)		
	Decline in belongingness	1 (2.4)	4 (6.3)	1 (1.9)		
Efforts to overcome the generation gap	Yes	33 (78.6)	47 (74.6)	33 (61.1)	2.08	0.128
	No	9 (21.4)	16 (25.4)	21 (38.9)		
If yes, how	Acknowledge differences	26 (61.9)	38 (60.3)	25 (46.3)	0.84	0.436
	Think of the other	6 (14.3)	4 (6.3)	6 (11.1)		
	Space for communication	1 (2.4)	5 (7.9)	2 (3.7)		
Work satisfaction	Dissatisfied	3 (7.1)	2 (3.2)	2 (3.7)	0.04	0.962
	Neutral	17 (40.5)	26 (41.3)	22 (40.7)		
	Satisfied	22 (52.4)	35 (55.5)	30 (55.6)		

OR, operating room; ER, emergency room; ICU, intensive care unit; CN, charge nurse; HN, head nurse.

#### 3.1.1. Generation X

All respondents from Generation X were female (100%, *n* = 42), with a mean age of 47.43 (±2.7) years. Most respondents were married (*n* = 38, 90.5%). Most nurses worked in outpatient care (*n* = 16, 38.1%), followed by internal medicine and surgery (*n* = 12, 28.6%). More than half of the nurses were full-time employees (*n* = 23, 54.8%). Relationship with co-workers was the most important factor considered in a job (*n* = 18, 42.9%), followed by compensation (*n* = 10, 23.8%), recognition (*n* = 7, 16.7%), having weekends off (*n* = 4, 9.5%), and regular working hours (7.1%, *n* = 3).

The nurses had an average of 287.55 (±36.3) months of clinical experience and 98.8 (±101.2) months of experience working in their current department. Of the 42 nurses, 92.9% (n = 39) responded that they experienced generational differences in their workplace, out of which 61.5% (*n* = 24) responded that they experienced conflicts with co-workers due to this. Having a different mindset was the most common source of conflict (62.5%, *n* = 15), followed by different communication styles (25.0%, *n* = 6), and differences in work culture (12.5%, *n* = 3).

Of the 39 nurses who reported experiencing generational differences in their workplace, 33.3% (*n* = 13) responded that such differences negatively affected their work. Lack of communication was the most common negative outcome of generational differences (69.2%, *n* = 9).

Of the 42 nurses from Generation X, 78.6% (*n* = 33) believed that efforts to overcome generational differences were necessary. Acknowledging one another’s differences was the most common way to overcome workplace conflict (61.9%, *n* = 26), followed by empathy (14.3%, *n* = 7). Among the Generation X nurses, 7.1% (*n* = 3) were dissatisfied with their current departments, 40.5% (*n* = 17) were neutral, 42.9% (*n* = 18) were satisfied, and 9.5% (*n* = 4) were very satisfied.

#### 3.1.2. Generation Y

The mean age of the nurses in Generation Y was 35.57 (±3.8) years, and most of them were female (95.2%, *n* = 60). Overall, 60.3% (*n* = 38) were married and 39.7% (*n* = 25) were unmarried. Internal medicine was the most common department (25.4%, *n* = 16), followed by the operating room (23.8%, *n* = 15), and intensive care unit (15.9%, *n* = 10). Of the 63 nurses in this generation, 82.5% (*n* = 52) worked three shifts, and 15.9% (*n* = 10) were full-time employees. All participants were general nurses (100%, *n* = 63). High compensation (50.8%, *n* = 32) was the most important factor for considering a job, followed by peer relationships (19.0%, *n* = 12), regular working hours (15.9%, *n* = 10), recognition (7.9%, *n* = 5), and having weekends off (6.3%, *n* = 4). The nurses had an average of 154.11 (±48.8) months of clinical experience and 84.24 (±67.7) months of work experience in their current department.

Of Generation Y nurses, 73.0% (*n* = 46) reported generational differences in their workplaces. Of these, 60.9% (*n* = 28) reported experiencing conflicts with co-workers, owing to this. Different mindsets were the most common reason for conflicts (64.3%, *n* = 18).

Of the 46 nurses who reported generational differences in their workplaces, 54.7% (*n* = 25) responded that generational differences negatively affected their work. A lack of communication and reduced motivation were the most common negative outcomes of generational differences (56%, *n* = 14), followed by reduced concentration and loyalty at work (32%, *n* = 6).

Of the 63 nurses from Generation Y, 74.6% (*n* = 47) responded that efforts to overcome generational differences were necessary. Acknowledging each other’s differences was the most common way to resolve conflicts arising from generational differences (60.3%, *n* = 38). Overall, 49.2% (*n* = 31) were satisfied with their current departments, 41.3% (*n* = 26) were neutral, 6.3% (*n* = 4) were very satisfied, and 3.2% (*n* = 2) were dissatisfied.

#### 3.1.3. Generation Z

The mean age of the nurses in Generation Z was 25.65 (±1.7) years; 83.3% (*n* = 45) were female, and 16.7% (*n* = 9) were male. Most of the nurses were unmarried (96.3%, *n* = 52). Internal medicine was the most common department (25.9%, *n* = 14), followed by the emergency room (22.2%, *n* = 12) and operating room (18.5%, *n* = 10). Most nurses worked three shifts (94.4%, *n* = 51), and all were general nurses (100.0%, *n* = 54). High compensation was the most important factor when considering a job (48.1%, *n* = 26), followed by peer relationships (31.5%, *n* = 17), and having weekends off (13.0%, *n* = 7).

The nurses had an average of 28.63 (±18.1) months of clinical experience and 26.04 (±17.7) months of work experience in their current departments. Among the 54 nurses from Generation Z, 46.3% (*n* = 25) reported generational differences in their workplace. Of these, 40% (*n* = 10) reported experiencing conflict with co-workers because of this.

Of the 25 nurses who reported generational differences in their workplace, 40% (*n* = 10) responded that these differences negatively affected their work. Lack of communication (40%, *n* = 4) was the most reported negative outcome from generational differences.

Among the 54 nurses, 61.1% (*n* = 33) believed that efforts to overcome generational differences were necessary. Acknowledging differences was the most common method of resolving generational conflicts (46.3%, *n* = 25%), followed by thinking about being in another’s shoes (11.1%, *n* = 6). Approximately 48.1% (*n* = 26) of the nurses from this generation were satisfied with their current departments, 40.7% (*n* = 22) were neutral, 7.4% (*n* = 4) were very satisfied, and 3.8% (*n* = 2) were dissatisfied or very dissatisfied.

### 3.2. Generational differences in general characteristics among nurses

Significant differences in demographic, occupational, and generational characteristics were found among Generations X, Y, and Z (Table 1).

Regarding demographic characteristics, Generations X, Y, and Z displayed significant differences in marital status (*F* = 72.28, *p* < 0.001), sex (*F* = 5.56, *p* = 0.005), and monthly income (*F* = 119.80, *p* < 0.001). Employment type (*F* = 41.69, *p* = <0.001) and job title (*F* = 85.57, *p* = <0.001) also indicated significant differences. The percentage of respondents who reported generational differences in their workplaces differed significantly across generations (*F* = 25.68, *p* < 0.001). Approximately 92.9% (*n* = 39) of the nurses from Generation X, 34.5% (*n* = 28) from Generation Y, and 46.3% (*n* = 25) from Generation Z reported generational conflicts; Generation X had the highest proportion of nurses who experienced generational differences.

There were significant differences in the percentage of nurses who experienced conflict with co-workers due to generational differences in their workplace (*F* = 15.37, *p* = <0.001). The types of generational conflicts differed across generations (*F* = 3.80, *p* = 0.025). For Generation X, having a different mindset was the most common reason for generational conflicts (62.5%, *n* = 15), followed by different communication types (25.0%, *n* = 6), and different work styles (12.5%, *n* = 3). For Generation Y, having a different mindset was reported as the most common reason for generational conflicts (64.3%, *n* = 18), followed by different communication types (14.2%, *n* = 4) and differences in the level of importance placed on work–life balance (10.7%, *n* = 3). For Generation Z, having a different mindset was the most common reason for generational conflict (50%, *n* = 5), followed by different work styles (20%, *n* = 2). Significant differences were also found in the negative outcomes of generational differences in the workplace between groups (*F* = 11.52, *p* < 0.001).

### 3.3. Generational differences in occupational values, communication types, job satisfaction, and organisational commitment

Table 2 presents the results of the analysis regarding occupational values, communication types, job satisfaction, and organisational commitment.

**Table 2:** Level of occupational values, communication types, job satisfaction, and organisational commitment (*N* = 159).

Variables	Total (*n* = 159)	Generation X (*n* = 42)	Generation Y (*n* = 63)	Generation Z (*n* = 54)
	N (%) or M ± SD	N (%) or M ± SD	N (%) or M ± SD	N (%) or M ± SD
Occupational values				
Extrinsic	94 (59.1)	20 (47.6)	39 (61.9)	35 (64.8)
Intrinsic	31 (19.5)	16 (38.1)	8 (12.7)	7 (12.7)
Extrinsic = Intrinsic	34 (21.4)	6 (14.3)	16 (25.4)	12 (22.2)
Communication types				
Rhetorical sensitivity	56 (35.2)	19 (45.2)	19 (30.2)	18 (33.3)
Rhetorical stubbornness	16 (10.1)	4 (9.5)	8 (12.7)	4 (7.4)
Rhetorical hypersensitivity	87 (54.7)	19 (45.2)	36 (57.1)	32 (59.3)
Job satisfaction				
Recognition and professional achievement	31.38 ± 3.80	31.30 ± 3.88	31.85 ± 3.82	30.88 ± 3.71
Maturity	20.89 ± 3.48	22.23 ± 2.75	20.79 ± 3.50	19.96 ± 3.68
Respect and recognition	29.67 ± 3.47	31.23 ± 2.89	29.03 ± 3.19	29.20 ± 3.84
Fulfilment of duty	14.09 ± 2.31	14.21 ± 2.51	13.69 ± 2.25	14.46 ± 2.17
Demonstration of professional competence	9.62 ± 1.58	10.21 ± 1.12	9.76 ± 1.49	9.00 ± 1.79
Job security and rewards	9.53 ± 1.90	10.19 ± 1.89	9.82 ± 1.62	8.68 ± 1.96
Total	115.20 ± 13.48	119.40 ± 12.03	114.96 ± 13.30	112.20 ± 14.13
Organisational commitment				
Value immersion	12.89 ± 2.56	14.02 ± 3.26	12.74 ± 2.14	12.20 ± 2.11
Commitment to effort	14.71 ± 1.97	14.83 ± 1.51	14.68 ± 1.79	14.66 ± 2.46
Retention commitment	13.18 ± 2.47	14.04 ± 2.08	13.14 ± 2.31	12.55 ± 2.77
Total	40.79 ± 5.89	42.90 ± 6.09	40.57 ± 5.40	39.42 ± 5.94

Extrinsic values were the most favoured for all three generations:47.6%, 61.9%, and 64.8% in Generations X, Y, and Z, respectively. Intrinsic values were favoured the most in Generation X (38.1%) and the least in Generation Y and Z (12.7% each). Overall, 25.4%, 22.2%, and 14.3% of nurses from the respective generations (Generations X, Y, and Z, respectively) equally favoured extrinsic and intrinsic values.

Regarding communication types, rhetorical reflectors were most common in Generation Y (57.1%) and Z (59.2%). There was an equal percentage of rhetorical-reflectors and rhetorical-sensitive types in Generation X (45.2%). Noble selves were the least common among the three generations.

In an analysis of the subdomains of job satisfaction, recognition from an organisation and professional achievements were rated as most important in all three generations [31.30 (±3.88), 31.85 (±3.82), and 30.88 (±3.71), for Generations X, Y, and Z, respectively]. The next most important subdomain was respectful interpersonal relationships [31.23 (±2.89), 29.03 (±3.19), and 29.20 (±3.84), for Generations X, Y, and Z, respectively]. Generations X and Z rated job security and sense of achievement as the lowest at 10.19 (±1.89) and 8.68 (±1.96), respectively. Generation Y rated applying professional skills the lowest [9.76 (±1.49)]. Generation X scored the highest in job satisfaction [119.40 (±12.03)], followed by Generation Y [114.96 (±13.30)], and Generation Z [112.20 (±14.13)].

In an analysis of the subdomains of organisational commitment, the highest scores were observed for effort commitment:14.83 (±1.51), 14.68 (±1.79), and 14.66 (±2.46) for Generations X, Y, and Z, respectively. Retention commitment and value commitment were the next highest types of commitment. Generation X had the highest organisational commitment score [42.90 (±6.09)], while Generation Z had the lowest [39.42 (±5.94)].

### 3.4. Generational differences in occupational values, communication types, job satisfaction, and organisational commitment

One-way ANOVA was used to examine generational differences in occupational values, communication types, job satisfaction, and organisational commitment (Table 3). Significant differences were found in job satisfaction across generations (*F* = 3.492, *p* = 0.303), and Generation X had higher job satisfaction than Generation Y. Furthermore, significant differences in organisational commitment were found across generations (*F* = 4.371, *p* = 0.010), with Generation X having a significantly higher organisational commitment than Generation Y. No significant differences in occupational values or communication types were found among the three generations.

**Table 3:** Generational differences in occupational values, communication types, job satisfaction, and organisational commitment (*N* = 159).

	Occupational values			Communication types			Job satisfaction			Organisational commitment		
	M ± SD	F (*p*)	Post-hoc	M ± SD	F (*p*)	Post-hoc	M ± SD	F (*p*)	Post-hoc	M ± SD	F (*p*)	Post-hoc
Generation X^a^	31.04 ± 3.39	0.765 (0.47)		90.14 ± 7.88	1.744 (0.18)		119.60 ± 12.03	3.492^*^ (0.03)	a > b > c	42.90 ± 6.10	4.371^*^ (0.01)	a > b > c
Generation Y^b^	30.73 ± 2.02			90.71 ± 6.60			114.96 ± 13.30			40.57 ± 5.40		
Generation Z^c^	31.42 ± 3.00			88.27 ± 7.40			112.20 ± 14.13			39.42 ± 5.94		

* *p* < 0.05. Post-hoc Scheffe’s test test (a > b > c).

### 3.5. Correlations between occupational values, communication types, job satisfaction, and organisational commitment across generations

Table 4 presents the results of the Spearman’s correlation analysis of occupational values, communication types, job satisfaction, and organisational commitment across the three generations. In Generation Z, no significant correlations were found between occupational values and communication, or between communication and job satisfaction. Organisational commitment significantly correlated with job satisfaction (*r* = 0.868, *p* = 0.001).

**Table 4:** Correlations between occupational values, communication types, job satisfaction, and organisational commitment across different generations (*N* = 159).

	Occupational values (1)	Communication types (2)	Job satisfaction (3)	Organisational commitment (4)
	*r* (*p*)	*r* (*p*)	*r* (*p*)	*r* (*p*)
Generation X (*n* = 42)				
1				
2	0.172 (0.276)			
3	0.060 (0.705)	0.098 (0.538)		
4	0.183 (0.245)	0.081 (0.609)	0.868^**^ (0.001)	
Generation Y (*n* = 63)				
1				
2	0.193 (0.130)			
3	0.435^**^ (0.001)	0.253^*^ (0.046)		
4	0.281^*^ (0.026)	0.107 (0.403)	0.734^**^ (0.001)	
Generation Z (*n* = 54)				
1				
2	0.264 (0.054)			
3	0.511^**^ (0.001)	0.024 (0.861)		
4	0.227 (0.098)	0.055 (0.695)	0.574^**^ (0.001)	
Total (*n* = 159)				
1				
2	0.197^*^ (0.013)			
3	0.357^**^ (0.001)	0.103 (0.196)		
4	0.122 (0.126)	0.040 (0.615)	0.727^**^ (0.001)	

* *p* < 0.05, **p < 0.01, ***p < 0.001.

In Generation Y, the most significant correlations were found between occupational values and job satisfaction (*r* = 0.435, *p* = 0.001) and between job satisfaction and organisational commitment (*r* = 0.734, *p* = 0.001). Furthermore, significant correlations were found between occupational values and organisational commitment (*r* = 0.281, *p* = 0.026) and between communication and job satisfaction (*r* = 0.253, *p* = 0.046).

In Generation Z, significant correlations were found between occupational values and job satisfaction (*r* = 0.511, *p* = 0.001), and between job satisfaction and organisational commitment (*r* = 0.574, *p* = 0.001).

Finally, the correlational analysis results for occupational values, communication types, job satisfaction, and organisational commitment for all three generations were combined. The most significant correlations were found between occupational values and job satisfaction (*r* = 0.357, *p* = 0.001) and between job satisfaction and organisational commitment (*r* = 0.727, *p* = 0.001). Occupational values were also significantly correlated with communication type (*r* = 0.197, *p* = 0.013).

## 4. Discussion

This study aimed to examine occupational values, communication types, job satisfaction, and organisational commitment among clinical nurses across generations. A nursing organisation consisting of members from different generations is subject to generational differences in communication methods, occupational values and behaviours, expectations of managers and the work environment, opinions about workplace power dynamics, and work styles (Stanley, 2010). Therefore, this study examined generational differences in occupational values, communication types, job satisfaction, and organisational commitment among clinical nurses. The study confirmed that to improve the quality of nursing care, it is necessary to consider developing various intervention programs based on the generation gap among clinical nurses, to reduce conflicts due to the generation gap, and ultimately establish the necessary system through a mutual understanding of education.

Generational conflicts are inevitable for nurses as they work in the rapidly changing environments of hospitals. Differences in beliefs and values formed from one’s experiences in social and cultural environments lead to generational conflicts, as individuals from different generations cannot understand one another (Park, 2017). However, no significant differences in occupational values were found among different generations in the current study. Extrinsic values were the most common among the three generations. This is consistent with a previous report stating that nurses perceive extrinsic occupational values as being more important than intrinsic ones (Ahn and Lee, 2020). Intrinsic values such as sense of achievement and applying one’s skills, aptitude, and interests were more important than extrinsic values such as compensation, financial stability, reputation, status, and work environment for all three generations (47.6%, 61.9%, and 64.8% for Generations X, Y, and Z, respectively). The extrinsic values were rated higher by Generations Y and Z than Generation X, which is consistent with a previous report that Generation Z tends to favour extrinsic values over intrinsic values (Kim and Han, 2013). This may be because, unlike Generation X, Generation Z has had to invest a large amount of time and effort in building an attractive resume and securing a job; therefore, they prioritise extrinsic values such as higher compensation (Lee H. S. and Kim, 2021; Lee Y. M. and Kim, 2021).

Nurses derive a sense of self-worth, pride, and achievement from their profession (Oh, 2019). In this study, Generation X rated a sense of achievement highly, whereas Generations Y and Z favoured compensation and the work environment over a sense of achievement. Additionally, Generation X rated intrinsic values higher than Generations Y and Z. While Generation X nurses learned to accept overtime and an excessive workload in a strict organisational culture, those from Generation Y and Z were less tolerant of long work hours and poor working conditions, often resorting to switching jobs (Lee H. S. and Kim, 2021; Lee Y. M. and Kim, 2021). This may be why intrinsic values were rated lower by Generation Y and Z than by Generation X. Ahn and Lee (2020) demonstrated that while nurses consider extrinsic values (e.g., financial compensation and job security) to be more important than intrinsic values (e.g., interest in the job, learning about oneself, and applying one’s skills), those from younger generations place a higher value on leisure, work–life balance, and interest in their job. In this study, the different generations exhibited different occupational values. Therefore, it is important to understand the generational differences in values. These results demonstrate the need to develop programs to help nurses set aside time for self-growth, cooperation, and respectful interaction with one another by understanding each other’s ways of thinking and values.

This study found no significant differences in the types of communication among the nurses. The rhetorically sensitive type is the most desirable and is characterised by its ability to communicate effectively. The noble self has strong opinions and projects these without hesitation (Park and Lee, 2013). A rhetorical reflector creates a sense of self, based on the current situation or the person they are talking to instead of following their own communication method, and has a strong tendency to avoid asserting their opinions for fear of upsetting others (Park and Lee, 2013). In this study, rhetorical sensitives and reflectors were equally common in Generation X (45.2%), whereas rhetorical reflectors were most common in Generation Y (57.1%) and Z (59.3%). There was a higher percentage of rhetorical sensitivity in Generation X (45.2%) than in Generations Y and Z (30.2% and 33.3%, respectively), which is inconsistent with a previous report of fewer rhetorical sensitivities in older generations (Park and Lee, 2013). Considering that rhetorical sensitivity refers to the trait of being sensitive to the situation and audience at hand (Ahn and Lee, 2020), and that nurses place high importance on taking care of others because of the nature of their jobs, the communication types observed in a nursing organisation may exhibit different characteristics from those of the general population in Generation X (Ahn and Yi, 2013).

Nurses from Generations Y and Z in South Korea tend not to assert their opinions, even in situations that warrant debate (Lee, 2007). Nurses from Generations Y and Z were more accustomed to authoritative communication methods when they were new to the job and the vertical hierarchical structure within hospitals. This explains why rhetorical reflectors may be more common in Generations Y and Z. Education to promote open and self-driven communication is necessary.

Significant differences in job satisfaction were found between the generations. Generation X scored significantly higher on job satisfaction than Generations Y and Z, possibly because Generation X had sufficient experience and knowledge to work independently, as opposed to nurses from Generation Z, who were less skilled, as they were more used to receiving instructions and supervision rather than working autonomously. Similarly, Al Maqbali (2015) reported lower job satisfaction among younger nurses. The nurses from Generation Z had the lowest job satisfaction, possibly because they had tighter work schedules as they worked in shifts and struggled to find time for hobbies and personal growth.

Generation X scored higher in the subdomains of job satisfaction, including personal maturation, respectful interpersonal relationships, applying professional skills, job security, and a sense of achievement, than other generations. Generation X placed more importance on intrinsic values than extrinsic ones, which is consistent with a study by Wilson et al. (2008) in which “baby boomers” had higher satisfaction associated with intrinsic values, such as compensation, promotion opportunities, company policies, authority, and peer relationships, compared with Generations X and Y. By contrast, Generation Y scored the lowest in respectful interpersonal relationships and task completion. Generation Y nurses play a crucial role in providing clinical care because they handle moderate to critically ill patients and are responsible for teaching junior nurses. The compensation they received did not match the importance or intensity of their tasks. Generations Z and Y, which are characterised by strong individualistic tendencies, experience a dilemma as they try to form respectful interpersonal relationships within the conservative and authoritative work culture of Generation X (Lee H. S. and Kim, 2021; Lee Y. M. and Kim, 2021). Generation Z scored the highest (14.46 ± 2.17) in completing tasks, which was one of the subdomains of job satisfaction. This indicates that Generation Z has a strong desire to accomplish their goals because they are exposed to a highly competitive environment, value objective evaluation and acknowledgement of their achievements, enjoy new challenges, and feel a sense of professional achievement upon completing their tasks (Wilson et al., 2008). Thus, medical institutions must implement measures to improve their work environment, and job satisfaction subdomains, such as compensation.

In this study, Generation X scored the highest in organisational commitment (42.90 ± 6.09 out of 60), while Generation Z scored the lowest (39.42 ± 5.94). This is consistent with previous studies, in which older nurses showed higher organisational commitment (Choi, 2014). Furthermore, Generation X had significantly higher organisational commitment than the other generations. This result supports a study by Ko (2004), in which nurses from Generation X had a higher organisational commitment than those from Generations Y and Z, as they were familiar with their hospital structure and administrative environment owing to sufficient clinical experience. This also suggests that nurses from Generation X increase their organisational commitment as their positions within their organisations become more secure over time. As nurses gain clinical experience, become familiar with hospital structures, and provide quality care in administrative environments, their organisational commitment increases. Thus, more experienced nurses are likely to have a higher organisational commitment. Additionally, effective communication with patients or other departments, gaining trust, and being acknowledged for their promotions and achievements are important factors in organisational commitment. In the case of Generation X, nurses may have an increase in organisational commitment as their positions within their organisations become more secure over time (Ko, 2004; Kim, 2017).

Oh (2019) reported lower organisational commitment among the younger generations, which is similar to the results of this study. Generation Z’s individualistic attitudes affect organisational commitment. An organisational culture that accepts individuals for who they are instead of criticising their traits or forcing them to change may help increase organisational commitment among Generation Z. A decrease in organisational commitment leads to staff turnover, and subsequently, hospital staff shortages. Nursing managers must recognise low organisational commitment among subordinates from Generation Z as a generational problem rather than an individual problem, and organisations must take measures to overcome old stereotypes and strengthen organisational commitment in the younger generations. Organisational commitment is more strongly associated with charismatic and transformational leadership than with intellectual stimulation or individual attention (Oh and Chung, 2011; Lee and Nam, 2016), indicating the importance of charismatic leadership exhibited by nursing managers. Nursing managers must show charisma by placing trust in their subordinates and presenting them with a vision rather than showing the traditional, authoritative type of charisma, which has been negatively perceived (Kim, 2018). Thus, senior nurses must try to form a supportive organisational culture through transformational leadership, and provide constructive criticism and support based on trust in their organisation. Specific strategies based on the interests of nursing organisations and generational characteristics are also needed to increase organisational commitment.

## 5. Conclusion

Organisational efforts to reduce cultural gaps between nurses from different generations and the development of a nursing workforce management program may positively transform a nursing organisation’s culture and improve its management. Intervention programs that consider generational differences among nurses should be developed based on these findings, in addition to policies and systems to reduce generational conflict and promote the appreciation of differences.

## Data availability statement

The raw data supporting the conclusions of this article will be made available by the authors, without undue reservation.

## Ethics statement

The studies involving human participants were reviewed and approved by Institutional Review Board of H University (HIRB-2022-048). The patients/participants provided their written informed consent to participate in this study.

## Author contributions

SL and JL: conception and design, provision of study materials or participants, data collection and intervention implementation, data analysis and interpretation, manuscript writing, and manuscript revision. All authors contributed to the article and approved the submitted version.

## Conflict of interest

The authors declare that the research was conducted in the absence of any commercial or financial relationships that could be construed as a potential conflict of interest.

## Publisher’s note

All claims expressed in this article are solely those of the authors and do not necessarily represent those of their affiliated organizations, or those of the publisher, the editors and the reviewers. Any product that may be evaluated in this article, or claim that may be made by its manufacturer, is not guaranteed or endorsed by the publisher.
